# Association between anger expression and attempted suicide at a general emergency hospital in the south of Brazil

**DOI:** 10.47626/2237-6089-2020-0097

**Published:** 2021-12-10

**Authors:** Cleonice Zatti, Sérgio Eduardo Silva de Oliveira, Luciano Santos Pinto Guimarães, Cleber Gibbon Ratto, Vitória Waikamp, Lucia Helena Machado Freitas

**Affiliations:** 1 Programa de Pós-Graduação em Psiquiatria e Ciências do Comportamento Universidade Federal do Rio Grande do Sul Porto Alegre RS Brazil Programa de Pós-Graduação em Psiquiatria e Ciências do Comportamento, Universidade Federal do Rio Grande do Sul (UFRGS), Porto Alegre, RS, Brazil.; 2 Departamento de Psicologia Clínica Universidade de Brasília Brasília DF Brazil Departamento de Psicologia Clínica, Universidade de Brasília (UnB), Brasília, DF, Brazil.; 3 Hospital de Clínicas de Porto Alegre Porto Alegre Brazil Hospital de Clínicas de Porto Alegre (HCPA), Porto Alegre, Brazil.; 4 Departamento de Educação Universidade LaSalle Canoas RS Brazil Departamento de Educação, Universidade LaSalle (UNILASALLE), Canoas, RS, Brazil.; 5 Departamento de Psiquiatria e Medicina Legal UFRGS Porto Alegre RS Brazil Departamento de Psiquiatria e Medicina Legal, UFRGS, Porto Alegre, RS, Brazil.

**Keywords:** Attempted suicide, anger, anger expression, anger traits

## Abstract

**Introduction:**

Suicide is one of the leading causes of death in the world. For every person who commits suicide, twenty or more have attempted to take their own lives. The emotional state of anger is often associated with suicidal behavior. However, this association needs to be further clarified.

**Objectives:**

This study sought to investigate the profiles of traits and expressions of anger in inpatients admitted to a general emergency hospital after surviving a suicide attempt.

**Methods:**

In this case-control study, a sample of 28 suicide survivors was matched for sex, age, and educational level with 56 controls. The State-Trait Anger Expression Inventory-2 was used to measure anger traits and expression.

**Results:**

Suicide survivors scored higher for anger traits and expression and lower for anger control than the control group. They also had lower levels of state anger and willingness to express anger verbally than the control group.

**Conclusions:**

Patients who attempted suicide and had high scores for anger expression (in and out) are inclined to have extreme difficulty in interpersonal relationships and rigidity towards change and are at higher risk of developing psychopathologies.

## Introduction

Suicide is an important contributor to mortality worldwide.^1^ Specifically, in Brazil, there is evidence^2^ that the country will not have been able to reduce the suicide rate by 10%, as proposed in the World Health Organization (WHO) Action Plan for 2020.^3^ This suggests that more efforts are needed to protect people from suicidal behavior, including designing prevention and intervention programs for suicide survivors. To provide a better health service to people with suicidal ideation, it is essential to identify as many psychological characteristics as possible that play a role in protection from or risk of suicidal behavior. The current study focuses on anger experiences in relation to suicide attempts.

Anger can be described according to two main components: state and trait.^4^ The former is an emotional experience involving negative feelings that can vary in intensity depending on the situational event, such as an injustice. The latter is a dispositional tendency to experience angry feelings as a personality trait. In both cases, anger is considered a transdiagnostic feature in several mental disorders,^5^ including suicidal behavior,^6 , 7^ and suicidal ideation.^8^ Shri et al.^9^ found a relationship between irritable mood and suicidal behavior in a thorough analysis of suicide attempts. Those who had attempted suicide were more likely to express aggressive traits than those who had not. In another study, Ammerman et al.^10^ investigated the role of anger in the incidence of suicidal and violent behavior in 2,295 undergraduate students aged 18 to 57 years. They found that trait anger was significantly associated with suicide attempts and violent behavior.

The current study arises from the need to seek a deeper understanding of feelings of anger in state and trait forms in individuals who have attempted suicide and those who have not. We are particularly interested in understanding the role of specific facets of anger in suicidal behavior. In view of the lack of studies on this subject in the literature, we hope our findings can improve prevention and health promotion practices. Thus, this study was designed, first, to assess whether there are differences in measures of anger expression in hospitalized patients who have attempted suicide compared to those who have not and, second, to evaluate aspects of anger as a risk factor for suicidal actions.

## Methods

### Participants and procedures

A total of 84 patients at an emergency hospital in Rio Grande do Sul, Brazil, participated in this case-control study. Data were collected from patients who were hospitalized or were being treated at the emergency room of the Hospital de Pronto Socorro de Porto Alegre between August 20, 2015 and March 21, 2016. Convenience sampling was used. At the time of data collection, 37 potential participants were identified using data provided by the Data Processing Company of the Municipality of Porto Alegre. Nine of these patients were not included in this study because of one or more of the following criteria: age under 18, refusal to participate, unresponsive to verbal intervention, or active psychotic symptoms. The control group (n = 56), with no history of attempted suicide, was formed by matching the age and gender of the members of the case group. Data collection was performed by a researcher with experience in assisting patients with suicidal behavior. Data on the groups interviewed and the sample calculation have been described elsewhere by Zatti.^11^

### Ethical aspects

The study was registered on the Plataforma Brasil (CAAE 44823315.1.0000.5327) and was approved in 2015 by the Research Ethics Committee of Hospital de Clínicas de Porto Alegre and by the Porto Alegre Municipal Health Department (1.180.317, June 30, 2015).

### Instruments

#### Sociodemographic questionnaire

A general information questionnaire was prepared to obtain participant data on gender, age, years of education, income, marital status, and occupation.

#### State-Trait Anger Expression Inventory-2 (STAXI-2)

This instrument consists of 57 items grouped into six scales, five subscales, and one Anger Expression Index, totaling 12 measures: State Anger (S-Ang); Feeling Angry (S-Ang/F); Feel Like Expressing Anger Verbally (S-Ang/V); Feel Like Expressing Anger Physically (S-Ang/P); Trait Anger (T-Ang); Angry Temperament (T-Ang/T); Angry Reaction (T-Ang/R); Anger Expression-Out (AX-O); Anger Expression-In (AX-I); Anger Control-Out (AC-O); Anger Control-In (AC-I); and the Anger Expression Index (AX Index). The lower the score, the less intense the feelings of anger are, except for the AC-O and AC-I items, for which higher scores indicate greater control over anger. Original copies of the scale forms were purchased from a department specializing in psychological testing in Brazil. The results were coded and interpreted according to the STAXI-2 manual.^4^ This instrument assesses aggressiveness by measuring experiences and expressions of anger and has good psychometric properties.^4^

## Data analysis

Quantitative data analysis was performed in PASW (SPSS) version 18. The sociodemographic characteristics of the sample have been presented elsewhere by Zatti et al.^12^

A generalized estimating equation (GEE) model^13 , 14^ was used to compare the mean scores for each STAXI-2 factor between groups. This model was used due to the sample pairing method (1 case for 2 controls of the same gender and age). In this study, the most appropriate analysis should consider each trio as a unit. Data are expressed as estimated means and confidence intervals. The model was constructed using a gamma distribution with log binding function, an exchangeable working correlation matrix, and a robust estimator covariance matrix.

A conditional logistic regression model was used to calculate the odds ratio of each STAXI-2 factor as a predictor of membership of the case group. In this model, participants from different groups (case or control) are only compared within the same matched set, which determines the conditional likelihood. Given the small sample size, we built four models, as follows: in model 1 the covariates we included were the scores of the three factors related to the state anger domain; in model 2 the covariates we included were the scores of the two factors of the trait anger domain; in model 3 the covariates we included were the scores of the four factors related to the anger expression and control domains; and, finally, in model 4 we used the scores at the domain level.

A p-value lower than 0.05 was considered statistically significant.

## Results

### Sample description

The gender distribution in the total sample (n = 84) was balanced (male = 46.4% [n = 39]; female = 53.6% [n = 45]) and the mean age was 35.6 years (standard deviation [SD] = 12.8; minimum = 19 and maximum = 71 years). The mean educational level was 9.9±4.6 years, and half of the sample had a monthly income over R$ 2,000 (interquartile range = [1.260-3.650]). A total of 61.9% (n = 52) reported being employed. The groups were matched for age and gender, and the mean educational levels (in years) were equivalent (cases: 9.7, SD = 4.0; controls: 10.1, SD = 4.9; *t* test; p = 0.707). There were no significant differences regarding income (Mann-Whitney test; p = 0.611), occupation (chi-square test; p = 0.522), or marital status (chi-square test; p = 0.177).

Twenty-eight individuals who attempted suicide were interviewed, and the methods they reported using included: 17 (61%) ingestion of medication; 3 (11%) each of a) jumping in front of a moving car or from a height; b) wrist/neck cutting; and c) poisoning (caustic soda, mothballs, rat poison); 2 (7%) each of a) hanging; b) burning; c) and illicit drug overdose; and 1 (3.5%) weapon/firearm injury, totaling 32 responses because more than one method was used in 4 cases. When patients in the case group were asked about recent and significant losses (bereavement or marital separation), 9 (32%) responded positively. Another important factor was recent marital separation, which had happened to 7 respondents (25%). Ten (36%) of the people who had attempted suicide reported suffering an intense loss in childhood.

### Comparison of anger levels between control and case groups

To identify the profile of anger experiences of suicide survivors, a GEE model was used to compare STAXI-2 factor scores between the control and case groups. Table 1 shows the results of these comparisons. In general, the means estimated were statistically different between groups, except for the factors State Anger and Feel Like Expressing Anger Physically (GEE models; p > 0.05). Interestingly, the State Anger p-value was borderline (GEE models; p = 0.053). In general, these results indicate that the participants in the case group tended to experience anger states more frequently, in addition to being more prone to irritability and having more problems with expression and control of anger than the participants in the control group.

**Table 1:** Comparison of estimated means of STAXI-2 subdomains between groups

Variable	Controls (n = 56)	Cases (n = 28)	p*

	Estimated mean (95% CI)	Estimated mean [95%CI]	
State anger	67.1 (61.6-73.2)	76.9 (70.6-83.8)	0.053
Feeling angry	71.0 (65.7-76.7)	81.1 (75.3-87.3)	0.044
Feel like expressing anger verbally	76.1 (74.3-78.0)	80.8 (76.9-85.0)	0.029
Feel like expressing anger physically	86.5 (85.6-87.5)	88.0 (86.2-89.7)	0.157
Trait anger	47.6 (39.5-57.5)	72.4 (62.2-84.4)	< 0.001
Angry temperament	56.7 (49.0-65.6)	80.1 (72.7-88.3)	< 0.001
Angry reaction	41.0 (32.6-51.7)	61.9 (49.6-77.2)	0.010
Anger Expression Index	43.7 (35.4-54.0)	73.7 (64.8-83.8)	< 0.001
Anger expression-out	61.1 (54.9-68.1)	77.1 (69.0-86.3)	0.001
Anger expression-in	56.1 (48.3-65.1)	76.6 (69.7-84.3)	0.001
Anger control-out	60.9 (53.0-69.9)	36.6 (28.3-47.3)	< 0.001
Anger control-in	64.4 (56.5-73.4)	42.8 (33.9-54.1)	< 0.001

95%CI = 95% confidence interval.

* Generalized estimating equation model – group factor analysis.

The patients’ high scores on the state anger scales indicate that they were feeling angry at the time of data collection (S-Ang/F) and that they were probably willing to express their anger verbally (S-Ang/V) at that time. The design of the present study did not enable identification and characterization of the type of anger experienced by these patients during data collection. However, according to observations, the case group tended to be momentarily angrier than the control group when the data were being collected.

The case group scored higher for the Trait Anger factor (72.4; SE = 5.6) than the control group (47.6; SE = 4.6) (GEE model; p < 0.001). People with high scores for this factor often experience feelings of anger and continually feel wronged by others or tend to feel more frustrated. The results showed that the case group had a greater tendency to experience feelings of anger and irritation (T-Ang/T), as well as a greater likelihood of experiencing anger in response to frustrating events (T-Ang/R) than the control group (p < 0.001 and p = 0.010, respectively).

Regarding the ability to express anger, the results showed that the participants in the case group tended to express anger at a higher level than those in the control group, both against others and against themselves. In fact, the estimated mean values of the AX-O and AX-I subscale T-scores were above 75, indicating 2.5 SD above the mean. In addition to this result, the case group’s ability to control anger was significantly lower than that of the control group (AC-O and AC-I; p < 0.001). Together, these results suggest that patients who attempted suicide tended to have no control over their anger, which resulted in chronic anger and increased risk of self-harm.

### Odds ratio of anger experiences as predictors of membership of the suicide survivors group

Conditional logistic regression models were used to identify the specific contribution of anger variables to prediction of people having suicidal behavior. Table 2 shows the odds ratios of the STAXI-2 factors for prediction of membership of the case group. The covariates in model 1 were the facets of the state anger domain. None of the facets from this domain made a statistically significant contribution to prediction of participants who had survived suicide. The covariates in model 2 were the facets of the trait anger domain. The results indicated that for each additional point on the angry temperament scale, the chance of a person being classified as belonging to the case group increased by 4.3%. The covariates in model 3 were the facets of the anger expression and anger control domain. As shown in Table 2 , the anger facet that statistically contributed to identifying people who had survived suicide was feeling anger expressed against oneself. A one-point increase in the score on the Anger Expression-In scale increased the chance of a person being classified as belonging to the case group by 3.6%. Finally, at the domain level, in model 4 we observed that the Anger Expression Index was the variable that most contributed to predicting membership of the case group. A one-point increase in the score on the Anger Expression Index scale increased the chance of a person being classified as belonging to the case group by 5.3%.

**Table 2:** Odds ratios for anger facets and domains as predictors of suicide survivors

	OR	(95%CI)	p*
Model 1 - State anger domain			
Feeling angry	1.018	(0.98-1.05)	0.291
Feel like expressing anger verbally	1.026	(0.96-1.10)	0.475
Feel Like expressing anger physically	1.021	(0.89-1.17)	0.760
Model 2 - Trait anger domain			
Angry temperament	1.043	(1.01-1.08)	0.010
Angry reaction	1.003	(0.99-1.02)	0.709
Model 3 - Anger expression/control domain			
Anger expression-out	1.013	(0.98-1.04)	0.397
Anger expression-in	1.036	(1.01-1.07)	0.015
Anger control-out	0.993	(0.96-1.03)	0.711
Anger control-in	0.967	(0.93-1.00)	0.081
Model 4 - Anger domains			
State anger	1.003	(0.97-1.04)	0.873
Trait anger	10.014	(0.99-1.04)	0.226
Anger Expression Index	1.053	(1.01-1.09)	0.007

* Conditional regression. The control group is used as reference.

95%CI = 95% confidence interval; OR = odds ratio.

## Discussion

Our study aimed to understand the role of specific facets of anger in suicidal behavior. To do so, we evaluated the levels of different anger experiences in a group of 28 patients admitted to a Brazilian emergency hospital due to a suicide attempt and then we compared them to a matched control group. The results indicate that the case group presented more problems related to anger than the control group, which is consistent with the literature.^15 , 16^ However, the current study provides further information about the anger profile of people who have attempted suicide.

As mentioned earlier, anger can be experienced as an emotional state or as a personality trait. The form of one’s experience and how one interprets different stimuli as frustrating and/or provocative will also depend on individual tendencies, thus affecting how one manages anger impulses.^4 , 17^ These experiences of anger are related to suicidal behavior. In the current study, we found that the two facets of anger that contribute most to identifying survivors of suicide were angry temperament and anger expressed against oneself.

Angry temperament is a facet of the trait anger domain and proved to be an important psychological characteristic of people who had survived a suicide attempt. This result allows us to infer that patients who have attempted to take their own lives are highly sensitive to criticism, outrage, and negative evaluations. In such situations, they experience intense feelings of anger and have greater difficulty containing their aggressive impulses. The Trait Anger domain has been associated with developing health problems such as coronary artery disease,^18^ binge eating disorder,^19^ bipolar and depressive disorders,^20^ alcohol and nicotine dependence,^21 , 22^ and increased suicide risk. Specifically regarding the angry temperament facet, the result found in the current study indicates that clinicians must pay thorough attention to this feature. Angry temperament, defined in this study as a tendency to experience anger quickly and with little provocation,^4^ has proved to be a crucial risk factor for suicide attempts. In fact, a high level of angry temperament has also been shown to be a predictor of aggressive behavior in a sample of patients who attempted suicide.^23^ The same authors also observed that angry temperament was a predictor of violent suicide attempts. It is known that, in general, patients hospitalized for attempted suicide tend to present high levels of aggressive profiles,^24^ with some differences by gender.^7^ This leads us to think that people with angry temperaments are at high risk for suicidal behavior because their anger effects are easily elicited.

The other anger facet that contributed to predicting patients who have attempted suicide was anger expressed against oneself. We highlight the potentially harmful combination of these two facets (angry temperament and anger expressed against oneself) with regards to suicidal behavior. Together, these facets lead us to infer that people who have angry temperament, who tend to be constantly irritated, and who express this anger against themselves may find suicide to be a (poorly adaptive) resource for handling the negative affects inherent to this psychological functioning profile. In fact, high levels of expression of anger against oneself proved to be a predictive factor of impulsive suicide attempts.^23^

Anger can facilitate suicidal behavior through an individual’s reduced ability to control or deal with the negative effects of anger.^10^ Individuals with a history of suicide attempts tend to have limited perceptions of emotions,^25^ which may partly explain suicidal behavior as an alternative for dealing with increased feelings of anger and as a potential mechanism for reducing the perceived negative effect.^10^ Self-directed anger tends to weaken emotional self-regulation skills and make it difficult for a person to calm down autonomously, and this can limit the individual’s ability to properly handle acute tendencies towards death.^7^ People with anger profiles may have difficulty dealing with acute urges to die by suicide when they arise.^7^ Emotional fragility makes people vulnerable to actively thinking about suicide or actually engaging in suicidal behavior.^26^

The psychological dynamics of people with angry temperaments, especially cases that express self-directed anger, seem to have some effect on suicide attempts.^23^ The act of trying to end life can be considered a nonverbal expression of anger due to a failure of intrapsychic communication.^15^ That is, self-directed anger in greater intensity, typical of unstable temperament, increases the chance of tension discharge in one’s body, which increases the risk of suicide. Psychological mediations are necessary so that anger is not discharged into the body as self-destructiveness. Through psychotherapeutic treatment, the patient can learn to regulate anger and understand emotions, which helps to prevent suicidal behavior. Functional anger management can act as a protective factor in life. Some reasons that prevent people from committing suicide are related to not wanting to harm their children or cause suffering to their families.^27^ It is essential to understand the role of protective factors for suicidal behavior to be able to increase reasons for living in people at risk of suicide.^28^

Some limitations should be considered in the present study. First, although a sample calculation was performed, the results of this cross-sectional study only allow reliable interpretations for the population in question. Also, the participants were hospitalized and the hospital is an environment to which they are not accustomed and this triggers negative feelings for many people, which may somehow have affected the results. Another limitation is related to the menstrual period, which is a factor that could influence women’s responses because of its effects on their emotional state. This was not controlled for in data collection. Taking these limitations into account, our findings may suggest that people who experience intense rage and have dysfunctional control of anger impulses are psychologically vulnerable to suicidal behavior.

## Conclusion

After surveying the literature, we conclude that the STAXI-2 instrument is still little used in research and clinical assessments. Given that anger influences the biological, psychological, and social aspects of a person’s life, anger measurement should be further explored. More direct studies on the development of rage in the family environment (bonding and parental conduct) are suggested. We suspect that better data on this topic could be of great importance for development of intervention and suicide prevention programs.
